# To Err Is Human, to Forgive Divine

**Published:** 1891-08

**Authors:** B. Fincke

**Affiliations:** Brooklyn, N. Y.


### TO ERR IS HUMAN, TO FORGIVE DIVINE.

B. FINCKE, M. D., BROOKLYN, N. Y.

My attention was drawn to a passage in my Refutation of Dr. Dudgeon's attack on the Hahnemannians and High Potencies (*Journal of Homœopathics*, vol. II, p. 272), as containing an error. There, Dr. Dudgeon is spoken of as one who priding himself on his translation of the *Materia Medica Pura* has trimmed out what did not suit his fancy. Now, it is true that Dr. Dudgeon translated the six volumes of Hahnemann's *Reine Arzneimittellehre*, which is the first half of his *Materia Medica Pura*, as well as an English scholar is able to do.

But the passage was not meant for this translation, of which at the time I was not cognizant, but for his attitude in relation to the whole *Materia Medica Pura* which appears in a tolerably clear light from a passage in a discussion on the Index of the *Cyclopœdia*, published in the *Monthly Homœopathic Review*, November 1st, 1890, p. 673, as follows: "Dr. Dudgeon pointed out that a great deal of criticism had already been exercised by the compilers of the *Cyclopœdia*. A great many thousands of symptoms had been eliminated, and a great many hundred of provings or so-called provings had been refused admission on account of their impurity (hear! hear!) and altogether the *Cyclopœdia* had been criticized to a very great extent as those who examined it, carefully, would be able to perceive. He spoke with a certain knowledge (hear! hear!) having been a great deal associated with Dr. Hughes in the translation of cases, and knowing what a great condensation had been effected in many of the records."

This leaves no doubt that Dr. Dudgeon commits himself to a silent partnership with the firm of Hughes, Dake & Co., which has undertaken the task of burying out of sight the symptoms and provings contained in the *Materia Medica Pura* which do not suit their fancy, though he is not guilty of this error in regard to his translation of the six volumes of the *Hahnemannian Reine Arzneimittellehre*. How the error in the Refutation could have crept in is incomprehensible, and the more so, as the manuscript was submitted to the scrutiny of several of my colleagues before it was printed. Though an enemy, I trust that the Doctor will forgive this slip of the pen. If so, it may be expected even that the "unpleasant subject" (high potencies) will no more "constitute in his eyes—as it always has done—the plague-spot of Homœopathy" (*British Journal*, January, 1881), and that looking with the eyes of a scientific man upon the experience of the last sixty years in Europe and America in this relation, it will appear to him, as to the Hahnemannians, the dawn of a higher development of Homœopathy.

*Ceterum censeo, macrodosiam esse delendam.*

